# Identifying and Addressing Unmet Needs in Dementia: The Role of Care Access and Psychosocial Support

**DOI:** 10.1002/gps.70066

**Published:** 2025-03-27

**Authors:** Annelie Scharf, Bernhard Michalowsky, Anika Rädke, Fabian Kleinke, Stefanie Schade, Moritz Platen, Maresa Buchholz, Michelle Pfaff, Audrey Iskandar, Neeltje van den Berg, Wolfgang Hoffmann

**Affiliations:** ^1^ Deutsches Zentum für Neurodegenerative Erkrankungen (DZNE) Rostock/Greifswald Greifswald Germany; ^2^ Section Epidemiology of Health Care and Community Health Institute for Community Medicine University Medicine Greifswald Greifswald Germany

**Keywords:** Alzheimer’s disease, Camberwell Assessment of Need for the Elderly (CANE), dementia, elderly population, environmental, physical, psychological, and social domains, health services research, needs assessment, people with dementia, primary care, unmet needs

## Abstract

**Objectives:**

People with dementia often have various unmet care needs across physical, psychological, environmental, and social domains. There’s a need to explore the association between domains of unmet needs and characteristics of people with dementia. The aim of this paper was to describe the domains of unmet and met needs among community‐dwelling people living with dementia, focusing on the home environment, physical, psychological, and social areas, and to identify sociodemographic, clinical, and health‐related parameters associated with unmet needs.

**Methods:**

We analyzed the InDePendent trial’s baseline data of *N* = 417 people with dementia. The Camberwell Assessment of Needs for the Elderly (CANE) was used to identify needs. Descriptive statistics were used to evaluate the distribution of needs and Logistic and Poisson regression models to detect sociodemographic and clinical factors associated with unmet needs in the four need domains.

**Results:**

People with dementia were on average 80.6 years old, mostly female (56%) and mildly to moderately cognitively impaired (85%). 98.6% of the participants had at least one need, of which just over a third (36.5%) were rated as met and just under two‐thirds (63.5%) as unmet. Lacking a care grade (access to social care) and low education were found to be risk factors for the occurrence of unmet needs in almost all areas. Factors such as increased medication use (OR = 1.10 [95%CI 1.02 to 1.19]) and loneliness (OR = 2.51 [95%CI 1.44 to 4.36]) were associated with a higher likelihood of unmet environmental needs. Similarly, the absence of a caregiver (OR = 2.81 [95%CI 1.03 to 7.64]), lower social support (OR = 1.71 [95%CI 1.02 to 2.84]), and poor physical health (OR = 8.40 [95%CI 3.39 to 20.81]) correlated with unmet physical needs. Participants living alone demonstrated higher levels of unmet physical needs (*β* = 0.27 [95%CI 0.01 to 0.53]). Depression (OR = 2.13 [95%CI 1.10 to 4.08]), living alone (OR = 1.73 [95%CI 1.04 to 2.86]) and poor physical health (OR = 2.82 [95%CI 1.15 to 6.93]) significantly increased the risk of unmet psychological needs. Social needs are more likely to be unmet in females (OR = 1.88 [95%CI 1.05 to 3.37]). Sensitivity analyses showed the positive effects of regular General Practitioner (GP) visits on the fulfillment of social needs (*β* = −0.61 [95%CI −1.01 to −0.22]).

**Conclusion:**

Access to comprehensive care, for example, through a care grade, education and regular visits to the GP, is just as important for meeting needs in various areas as psychosocial measures aimed at reducing loneliness, living alone, and social exclusion. Both areas must be given equal consideration to improve the living and care situation of people with dementia sustainably.

**Trial Registration:** The study is registered as a clinical trial (ClinicalTrials.gov Identifier: NCT04741932). The study protocol is published elsewhere

**Summary**

- *High Prevalence of Unmet Needs*: The study reveals that nearly all people with dementia (98.6%) have at least one unmet need, with the majority (63.5%) experiencing unmet needs across environmental, physical, psychological, and social domains.
- *Sociodemographic Risk Factors*: Key factors associated with unmet needs, such as low educational attainment, the absence of a caregiver, and the lack of social care access by a care grade, significantly impact nearly all areas—particularly physical and social care—leaving people with dementia who lack formal access to care services with limited opportunities to address essential needs.
- *Psychosocial Risk Factors*: The study emphasizes the role of psychosocial factors like loneliness and living alone, which are strongly linked to unmet needs—inequalities that are further exacerbated by a lack of social support networks and formal care services.
- *Holistic Care Approach*: Meeting the care needs of people with dementia requires a comprehensive approach that integrates medical, nursing, and psychosocial interventions. Reducing loneliness, addressing social exclusion, and improving access to care are essential for enhancing their quality of life.

## 1 Introduction

As the population ages and demographic change progresses, the number of people with dementia is rising and is expected to more than double by 2050 [1, 2]. This creates a growing demand for efficient, individualized care [3]. In addition to physical problems, older people often also have psychological, environmental, social, and care issues, which result in a variety of unmet care needs [4, 5]. These unmet needs can place a heavy burden on social, economic, and health systems [6] because adequately managing the symptoms of dementia, comorbidities and quality of life requires a wide range of support and services [7].

A common way to identify unmet needs is to administer standardized tools, such as the Camberwell Assessment of Needs for the Elderly (CANE), to assess older people’s physical, psychological, social, and environmental needs [8]. Unmet needs can be defined as a situation in which a person has a significant problem that could be solved with the help of an intervention, such as assistance with activities of daily living like bathing or managing medications [9, 10]. Many studies used the CANE questionnaire in people with dementia to describe how many met and unmet needs exist in which areas of life [11, 12, 13, 14, 15, 16, 17, 18]. Some of them [11, 12, 17, 18] concluded that most unmet needs were related to memory, psychological distress, daytime activities, and social contacts/company. Other studies investigated the association of the number of unmet needs with various parameters [17, 18, 19, 20, 21, 22, 23, 24]. For example, a study from the UK concluded that unmet needs are associated with increasing behavioral problems, young age, depression and anxiety [21]. The quality of life [23, 25], as well as caregiver characteristics such as age, gender, education, and relationship also influence unmet needs [22]. The European ActifCare study examined how unmet need areas relate to quality of life and neuropsychiatric symptoms. Findings showed a link between unmet needs in “mobility/falls” and lower quality of life [26] and between “daytime activities” and “company” with more affective and psychotic symptoms [27].

Global changes in economic, social, and technological spheres are creating new, unexplored unmet needs in dementia care. These relatively unknown needs must also be recognized and resolved [28]. Only a few studies [26, 27] have investigated the individual domains of CANE and their correlation with relevant health‐related and other parameters in people with dementia. Therefore, this paper aims to expand the previous research results by describing the domains of unmet and met needs in a specific sample of community‐dwelling people with dementia, identifying sociodemographic, clinical, and health‐related parameters associated with unmet needs in the key areas home environment, physical, psychological, and social state. This comprehensive analysis will contribute to developing individualized, high‐quality, and effective care for people with dementia by identifying and addressing unmet needs.

## 2 Materials and Methods

### 2.1 Study Design, Setting and Recruitment

This cross‐sectional analysis was based on data from the German InDePendent trial (“Interprofessional Dementia Care: Redistribution of tasks between physicians and qualified nurses in primary care”), a network‐based, multi‐center, cluster‐randomized, controlled intervention study [29] evaluating extended nursing roles in community‐dwelling people with dementia and their caregivers in primary care compared with usual care.

Participants were recruited in the primary care setting by General Practitioners (GP) and specialists who were members of one of five physician networks in three federal states of Germany (Mecklenburg‐Western Pomerania, Brandenburg, and Hesse). Inclusion criteria for people with dementia were being community‐dwelling, screened positive for dementia (DemTect ≤ 8) [30] or being formally diagnosed with dementia, and provision of written informed consent. If the person could not provide written informed consent, their legal representative was asked to sign on their behalf, and caregivers were also invited to participate and provide consent. The ethics committees approved implementing the InDePendent study (registration number: BB 144/20; AS 81(bB)/2020; 2020‐2081‐zvBO). The recruitment period started January 2021, and ended in December 2022. The present analysis was based on baseline data.

### 2.2 Data Assessments

People with dementia and their caregivers received a standardized, computer‐assisted assessment via face‐to‐face interview at home by qualified nurses with extended nursing roles [29].

#### 2.2.1 Needs Assessment

Participants needs were identified using the German version of the Camberwell Assessment of Needs for the Elderly (CANE) as a self or proxy rating (completed by caregivers, if available) [8]. This assessment first asks whether there is a need in a certain domain and, if so, whether or not it has already been met. In the rare case (*n* = 10) that both the person with dementia and their caregiver responded to the CANE questionnaire, preference was given to the caregiver’s version. This choice was based on the observation that people with dementia often report considerably fewer (unmet) needs than their caregivers [8, 22, 31]. The questionnaire covers 25 different aspects of daily life, with an additional two items that specifically address the needs of caregivers (only people with dementia’s needs were considered in this analysis). Needs were rated as “met need” (problem receiving suitable assessment/intervention) or “unmet need” (problem requiring further assessment or no intervention resp. inappropriate intervention) [32].

We categorized the 25 CANE aspects based on previous work by Ploeg et al. [15] and Stein et al. [33] thematically into four categories: (1) environmental needs (living situation, housekeeping, diet, caring for someone else, financial situation, financial support), (2) physical needs (self‐care, seeing/hearing, mobility, falls, incontinence, physical diseases, medication), (3) psychological needs (memory, psychotic symptoms, psychological distress, deliberate self‐harm, inadvertent self‐harm, behavioral disorder, alcohol abuse), and (4) social needs (daytime activities, not being informed about health status, abuse/neglect, lacking social contacts, intimate relationships) [15, 33]. Additionally, unmet needs were totaled, resulting in a count variable (0–25 needs) (for sensitivity analysis).

#### 2.2.2 Sociodemographic and Clinical Variables

Sex, age, school education (up to 10 years = no qualification, elementary school/over 10 years = secondary school, high school, other), living situation (alone/not alone), self‐assessed financial situation (good/not good), and caregiver availability (yes/no) were assessed.

Cognitive impairment was assessed using the Mini‐Mental State Examination (MMSE), where lower scores indicate moderate to severe impairment (30 no hint for cognitive impairment, 29–20 mild, 19–10 moderate, ≤ 9 severe) [34, 35]. Functional impairment was measured by the Bayer‐Activities of Daily Living Scale (B‐ADL), where scores range from 1 (best) to 10 (worst) (< 3 good, 3–< 8 average, 8–10 poor) [36, 37]. Missing values of the people with dementia version were replaced by the caregiver information. Depression (self reported) was measured using the Geriatric Depression Scale (GDS) (0–5 no depressive symptoms, > 6 depression) [38, 39]. General health status was assessed using the EQ‐5D‐5L (self‐rating instrument), which covers five dimensions (mobility, self‐care, usual activities, pain/discomfort, anxiety/depression), each with five levels of response (no problems, slight problems, moderate problems, severe problems, and extreme problems) [40]. A health utility index was calculated using the German value set by Ludwig et al. [41], anchored between −0,6 (worst) and 1 (best) (categories: > 0.9 good, ≤ 0.9–0.5 average, ≤ 0.5 poor). Functional impairment was also measured by the presence of a long‐term care grade (“Pflegegrad”), a classification in Germany indicating the level of need for care and support due to functional and cognitive impairments, ranging from one to five. The care grade enables patients to receive and reimburse formal care services and home support and, therefore, can be seen as social care access.^1^ The body‐mass‐index (BMI) was categorized into: < 18.5 underweight, ≥ 18.5–24.9 normal weight, ≥ 25 pre‐obesity/obesity [42]. All ICD‐10 diagnoses documented in the treating practitioner’s records and all medications, including over‐the‐counter drugs, were assessed.

The utilization of care services (GP and neurologist/psychiatrist) within the last 3 months (yes/no) was measured using the Questionnaire for Health‐related Resources in Older People (FIMA) [43]. Missing values of the people with dementia version were replaced by the caregiver information. Social support (self reported) was measured using the F‐SozU survey (< 3 low, 3–< 4 average, > 4 high) [44]. Loneliness was recorded using The De Jong Gierveld short scales for emotional and social loneliness (DJGLS) (0–2 not lonely, 3–8 moderate, > 9 severe) [45, 46].

### 2.3 Statistical Analyses

Missing data on covariates were imputed using multiple imputations by chained equation (MICE). MICE imputes missing data iteratively, with each variable being imputed based on the observed values of others, and the model being updated in each iteration [47]. Using descriptive statistics, we summarized the variables describing the sample with no needs, met and unmet needs. Differences between those who had no needs and those who had needs were analyzed using Logistic regression models estimating Odds Ratios. We also used Logistic regression models to estimate the probability of unmet needs across need domains. Poisson regression models were carried out as sensitivity analyses to additionally identify the associations between the number of unmet needs and sociodemographic and clinical factors. Statistical analyses were performed using StataSE 16 (TX, USA: StataCorp. 2019).

## 3 Results

In total, 417 participants who completed the baseline assessment were, on average, 80.6 years old, primarily female (56%), mildly to moderately cognitively impaired (85%), not living alone (60%), and had a caregiver (93%) (Table 1).

**TABLE 1:** Sociodemographic and clinical variables of people with dementia.

	Total sample (*n* = 417)	
Sex (female), *n* (%)	233	55.9%
Age, mean (SD)	80.6	6.9
Having a care grade^a^ (yes), *n* (%)	279	66.9%
Living alone (yes), *n* (%)	167	40.1%
Caregiver availability (yes), *n* (%)	389	93.3%
Education		
Up to 10 years, *n* (%)	245	58.7%
Over 10 years, *n* (%)	172	41.3%
Financial situation (good), *n* (%)	339	81.3%
Cognitive impairment (MMSE)		
Score, mean (SD)	17.3	7.5
Mild, *n* (%)	186	44.6%
Moderate, *n* (%)	168	40.3%
Severe, *n* (%)	63	15.1%
Number of diagnoses, mean (SD)	10.8	9.3
Number of medications, mean (SD)	6.7	3.6
Body‐mass‐index		
Mean (SD)	26	4.4
Normal weight	182	43.7%
Underweight resp. pre‐obesity/obesity	235	56.3%
Quality of life (EQ‐5D‐5L)		
Score, mean (SD)	0.74	0.23
Good, *n* (%)	101	24.2%
Average, *n* (%)	255	61.2%
Poor, *n* (%)	61	14.6%
Functional impairment (B‐ADL)		
Score, mean (SD)	5.8	2.3
Good, *n* (%)	62	14.9%
Average, *n* (%)	273	65.5%
Poor, *n* (%)	82	19.7%
Depression (GDS)		
Score, mean (SD)	3.7	2.8
No depressive symptoms, *n* (%)	355	85.1%
Indication for depression, *n* (%)	62	14.9%
Utilization (FIMA)		
General practitioner (yes), *n* (%)	364	87.3%
Neurologist/Psychiatrist (yes), *n* (%)	127	30.5%
Social support (F‐SozU)		
Mean (SD)	3.9	0.5
Low/average, *n* (%)	258	61.9%
High, *n* (%)	159	38.1%
Loneliness (DJGLS)		
Mean (SD)	2.5	2.7
Not lonely, *n* (%)	278	66.7%
Moderate/severe, *n* (%)	139	33.3%
Unmet needs (CANE)—Both perspectives		
Mean (SD)	2.4	2.7

Abbreviations: B‐ADL, Bayer activities of daily living scale, range 0–10, lower score indicates better performance (categories: < 3 good, 3–< 8 average, 8–10 poor); EQ‐5D‐5L, range 0–1; higher score indicates better health‐related quality of life (categories: > 0.9 good, ≤ 0.9–0.5 average, ≤ 0.5 poor); F‐SozU, perceived social support questionnaire, range 0–5; higher score indicates better social support (categories: < 3 low, 3–< 4 average, > 4 high); GDS, geriatric depression scale, sum score 0–15, score ≥ 5 indicates depression (categories: 0–5 no depressive symptoms, > 6 depression); Loneliness, range 0–11; higher score indicates severe loneliness (categories: 0–2 not lonely, 3–8 moderate, > 9 severe); MMSE, mini mental state examination, range 0–30, higher score indicates better cognitive functioning (categories: 29–20 mild, 19–10 moderate, ≤ 9 severe); SD, standard deviation.

^a^ The long‐term care insurance provider in Germany uses five care grades to rate how independently a person can manage their everyday life and how severely affected their mental, psychological and physical abilities are. The greater the impairment, the higher the care grade and also the long‐term care insurance benefits.

### 3.1 Description of Needs

98.6% of *N* = 417 participants stated that they had at least one need, of which just over a third (36.5%) were rated as met and just under two‐thirds (63.5%) as unmet. An average of 2.1 (SD = 2.5) needs were indicated per participant.

Most *n* = 411 people with dementia had *needs* in memory (82.0%), housekeeping (73.9%), mobility (68.8%), drugs (63.3%), seeing/hearing (59.9%), and daytime activities domains (59.2%). Summarizing these needs into the four main areas, most participants had needs in the physical area (95.4%), of which half of the participants (43.2%) had unmet needs. A large proportion of the participants also had needs in the psychological (84.9%), environmental (88.0%), and social (72.2%) domains.

Most *unmet needs* were found in the areas of memory, daytime activities, financial support, social contacts, and psychological distress (Table 2).

**TABLE 2:** People with dementia with met and unmet needs, reported by people with dementia and caregiver (*n* = 417).

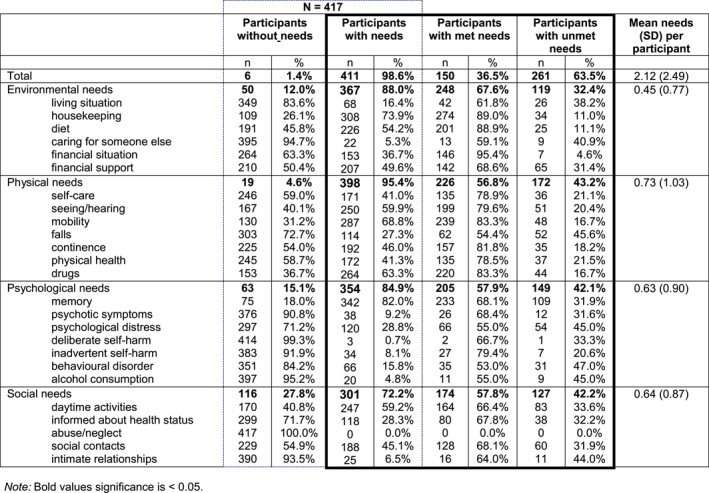

Abbreviations: SD, standard devition.

### 3.2 Differences Between Participants Without Versus With Needs

People with dementia with environmental (*n* = 367) (OR = 1.26 [95%CI 1.07 to 1.48]) and physical needs (*n* = 398) (B‐ADL) (OR = 1.40 [95%CI 1.10 to 1.85]) were in poorer physical condition compared to the ones without needs. Participants with psychological needs (*n* = 354) were more depressed (OR = 1.19 [95%CI 1.02 to 1.39]) and younger (OR = 0.94 [95%CI 0.89 to 0.98]) compared to those without needs in this area. Furthermore, participants with social needs (*n* = 301) had higher physical (OR = 1.26 [95%CI 1.21 to 1.42]) and cognitive deficits (OR = 0.94 [95%CI 0.90 to 0.98]) (Supporting Information S1: Table S1).

### 3.3 Association of Unmet Needs With Sociodemographic and Clinical Parameters

#### 3.3.1 Environmental Unmet Needs

People with dementia who do not have a care grade (OR = 3.38 [95% CI 1.95 to 5.87]), meaning they have not been formally assessed to qualify for care services, those taking more medications (OR = 1.10 [95% CI 1.02 to 1.19]), and those feeling lonely (OR = 2.51 [95% CI 1.44 to 4.36]) are associated with a higher likelihood of having unmet environmental needs. Contrary to this, a higher age (OR = 0.95 [95%CI 0.92 to 0.99]), and a higher number of diagnoses (OR = 0.95 [95%CI 0.91 to 0.98]) were correlated with a lower likelihood of having unmet needs. Sensitivity analysis confirmed these findings and showed a further association between low education and a higher number of unmet needs (*β* = 0.34 [95%CI −0.01 to 0.96]) (Supporting Information S1: Table S2).

#### 3.3.2 Physical Unmet Needs

Not having a caregiver (OR = 2.81 [95%CI 1.03 to 7.64]) and no care grade (OR = 3.02 [95%CI 1.81 to 5.04]), low social support (OR = 1.71 [95%CI 1.02 to 2.84]), poor physical condition (OR = 8.40 [95%CI 3.39 to 20.81]), and lower education (OR = 1.72 [95%CI 1.06 to 2.78]) increased the likelihood of having unmet physical needs. In contrast, moderate to severe cognitive impairment (OR = 0.58 [95%CI 0.35 to 0.97]) was associated with a lower likelihood of having unmet needs. These findings were confirmed in the sensitivity analysis that additionally revealed associations between living alone (*β* = 0.27, [95%CI 0.01 to 0.53]) and an increasing number of unmet needs and between better health‐related quality of life (*β* = −0.76 [95%CI −1.3 to−0.19]) and a lower number of unmet physical needs.

#### 3.3.3 Psychological Unmet Needs

Living alone (OR = 1.73 [95%CI 1.04 to 2.86]), lower education (OR = 1.86 [95%CI 1.12 to 3.08]), not having a care grade (OR = 2.50 [95%CI 1.48 to 4.24]), and a poor physical condition (OR = 2.82 [95%CI 1.15 to 6.93]) increased the likelihood of having unmet psychological needs. Also, the probability of having unmet needs in this area decreased with age (OR = 0.96 [95%CI 0.92 to 0.99]) and increased with depression (OR = 2.13 [95%CI 1.11 to 4.08]). The sensitivity analysis confirmed these findings, adding evidence for an association between the number of unmet psychological needs decrease and a higher BMI (*β* = −0.03 [95%CI −0.07 to −0.01]).

#### 3.3.4 Social Unmet Needs

The probability of having unmet social needs was higher in females (OR = 1.88 [95%CI 1.05 to 3.37]) and in those who had no care grade (OR = 4.66 [95%CI 2.28 to 8.73]). Overweight and underweight participants had lower odds of having unmet social needs (OR = 0.54 [95%CI 0.31 to 0.95]). The sensitivity analysis confirmed these findings and also showed a significant association between GP treatments during the last 3 months (*β* = −0.61 [95%CI −1.01 to−0.22]) and a lower number of unmet social needs, but also between recent neurologist treatment and a higher number of unmet needs (*β* = 0.35 [95%CI 0.03 to 0.68]). Also, a moderate to strong feeling of loneliness (*β* = 0.11 [95%CI 0.04 to 0.18]) and a better cognitive status (*β* = 0.02 [95%CI 0.01 to0.05]) were negatively associated with a higher number of unmet social needs. Associations between unmet needs and sociodemographic and clinical factors are shown in Table 3.

**TABLE 3:** Logistic regression models for met needs versus unmet needs.

	Unmet needs (CANE)							
	Environmental^a^		Physical^b^		Psychological^c^		Social^d^	
	OR	SE	OR	SE	OR	SE	OR	SE
Patient age (years)	0.95*	**0.01**	1.01	0.01	0.96*	**0.01**	0.96	0.01
Patient sex (Ref. male)	1.29	0.35	1.10	0.27	1.27	0.32	1.88*	**0.56**
Living alone (Ref. not alone)	0.87	0.24	1.54	0.38	1.73*	0.44	1.42	0.41
Caregiver availability (Ref. yes)	1.38	0.72	2.81*	**1.43**	0.75	0.38	1.94	1.18
Education (Ref. higher sec. education)	1.66	0.45	1.72*	**0.42**	1.86*	**0.47**	1.33	0.39
Financial situation (Ref. good)	1.40	0.45	0.94	0.28	1.65	0.50	1.25	0.45
GP visit last 3 months (Ref. no)	1.44	0.57	1.22	0.47	1.12	0.41	0.58	0.24
Neurologists visit last 3 months (Ref. no)	0.68	0.19	1.06	0.27	1.11	0.28	1.63	0.49
Functional impairment (B‐ADL) (Ref. good)								
Average	1.08	0.41	2.55*	**0.93**	1.67	0.61	1.11	0.53
Poor	1.63	0.78	8.40***	**3.88**	2.82*	**1.29**	1.98	1.09
Depression (GDS) (Ref. no depressive symptoms)								
Indication for depression	1.68	0.58	1.74	0.57	2.13*	**0.70**	1.73	0.66
Cognitive impairment (MMSE) (Ref. mild)								
Moderate/severe	0.85	0.24	0.58*	**0.15**	1.01	0.26	0.82	0.25
General health (EQ‐5D‐5L index) (Ref. good)								
Average	0.84	0.27	1.24	0.36	0.85	0.25	1.77	0.63
Poor	1.03	0.43	1.18	0.47	0.61	0.25	0.97	0.47
Body mass index (BMI, kg/m^2^) (Ref. normal weight)								
Underweight/pre‐obesity + obesity	0.55	0.14	0.72	0.17	0.76	0.18	0.54*	**0.15**
Having a care grade (Ref. yes)	3.38***	**0.95**	3.02***	**0.78**	2.50***	**0.67**	4.66***	**1.49**
Number of diagnoses	0.95*	**0.01**	0.99	0.01	0.97	0.01	1.01	0.01
Number of drugs taken	1.10**	**0.04**	1.05	0.03	1.03	0.03	1.01	0.04
Social support (F‐SozU) (Ref. high)								
Low/average	0.74	0.21	1.71*	**0.44**	1.26	0.34	1.43	0.44
Loneliness (Ref. not lonely)								
Moderate/severe	2.51**	**0.70**	0.89	0.23	1.34	0.35	1.48	0.43

*Note:* Bold values significance is < 0.05.

Abbreviations: B‐ADL, Bayer activities of daily living scale, range 0–10, lower score indicates better performance (categories: < 3 good, 3–< 8 average, 8–10 poor); CI, 95% confidence interval; EQ‐5D‐5L, range 0–1; higher score indicates better health‐related quality of life (categories: > 0.9 good, ≤ 0.9–0.5 average, ≤ 0.5 poor); F‐SozU, perceived social support questionnaire, range 0–5; higher score indicates better social support (categories: < 3 low, 3–< 4 average, > 4 high); GDS, geriatric depression scale, sum score 0–15, score ≥ 5 indicates depression (categories: 0–5 no depressive symptoms, > 6 depression); Loneliness, range 0–11; higher score indicates severe loneliness (categories: 0–2 not lonely, 3–8 moderate, > 9 severe); OR, odds ratio; Ref = reference.

^a^ Model: *n* = 367, Pseudo *R*2 = 0.1245, *p* < 0.0001.

^b^ Model: *n* = 354, Pseudo *R*2 = 0.0994, *p* = 0.0004.

^c^ Model: *n* = 398, Pseudo *R*2 = 0.1297, *p* < 0.0001.

^d^ Model: *n* = 301, Pseudo *R*2 = 0.1672, *p* < 0.0001.

*p < 0.05; **p < 0.01; ***p < 0.001 tested for each independent variable with the dependent variable (CANE).

## 4 Discussion

Our results confirm previous study findings [11, 12, 13, 14, 15, 16, 17, 18, 19, 20] showing a high disease burden among people with dementia, with nearly all participants having at least one need and about two‐thirds of these needs unmet. As in the literature [11, 12, 17, 18], most unmet needs are in psychological (memory and distress) and social domains (daytime activities, social contacts) and in managing finances. Psychological distress is prevalent due to dementia's nature [48] and can lead to loss of self‐esteem and social contact [3, 12]. A UK study found that people with dementia in care facilities spent 17% of their time sleeping, 33% doing basic activities (such as eating or going to the toilet), only 14% talking to others, and 30% of their time socially withdrawn [49]. Home‐based activities tailored to their interests can increase engagement, reduce behavioral symptoms, and relieve caregiver burden [50]. These findings highlight the urgent need for social inclusion interventions.

### 4.1 Association Between the Number of Unmet Needs and Sociodemographic and Clinical Parameters

#### 4.1.1 Environmental Unmet Needs

The likelihood of unmet environmental needs decreases by 5% with each additional *year of life*. This may be due to earlier diagnoses, enabling timely support. Furthermore, each additional *diagnosis* reduces the risk of unmet environmental needs by 5%, as multimorbid people receive more comprehensive care [51, 52] and are likely to be monitored more frequently. A Swiss study [51] found that for people over 65, each chronic condition led to 3.2 more consultations. This helps identify care needs, enabling targeted support for household tasks and finances. However, for each additional *medication* taken, the risk of unmet environmental needs increases by 10%. Incorrect intake of medication [53] or the increased likelihood of drug interactions [54] leads to a higher number of needs for the participants. A longitudinal analysis [55] of 352 people with dementia also showed that over half of the participants take at least one inappropriate medication, which can negatively impact their quality of life and increase hospitalizations. Regular medication reviews are crucial to prevent these risks. As shown in the literature [17, 56, 57], lower *education* is another factor associated with a higher number of unmet needs. Education is an enabling factor, improving access to information and support services, such as financial assistance [17, 56]. Additionally, participants without a *care grade* have limited access to social care, which is essential for addressing issues like housework or financial management. *Loneliness* is also a key contributor to unmet environmental needs. It often leads to mental health problems [58, 59] and social isolation, which reduces motivation to manage daily tasks. A dementia diagnosis can further stigmatize individuals, increasing social withdrawal [60] and limiting access to support networks. Loneliness may suggest that participants lack informal support from family caregivers or live alone, leaving them without assistance for tasks like navigating bureaucratic matters, such as applying for long‐term care benefits.

#### 4.1.2 Physical Unmet Needs

People with dementia who *live alone* and have lower levels of *education* face more unmet physical needs, consistent with previous findings [22, 61, 62]. People with dementia living alone are more affected by social isolation and inadequate social and medical care [61], making it difficult to manage physical needs such as vision, hearing, mobility, and medication management. This underscores the importance of promptly identifying people with dementia who live alone, allowing for regular monitoring and improved support from social services [61]. As *physical health* declines, the need for support increases, and the risk of unmet needs grows, as noted in previous studies [20]. Limited access to *care* services, like long‐term care benefits, can worsen this deterioration. Unmet physical needs increase as *cognitive status* improves, which seems counterintuitive at first glance, but this aligns with the literature [24, 56]. People with mild cognitive impairment often have their physical needs overlooked, especially in the early stages of dementia [24]. For this reason, there should be a focus on the early stages of dementia to influence the quality of care positively [63]. The literature [23, 24] and our sensitivity analysis show a link between better health‐related *quality of life* and fewer unmet needs. *Social* *support* reduces psychological and social unmet needs [20]. Those with better social inclusion have better access to care services, and the absence of a *caregiver* significantly increases unmet physical needs, especially in bureaucratic tasks such as applying for care assistance. Ensuring community participation and access to social workers or befriending services can help address these needs [20].

#### 4.1.3 Psychological Unmet Needs

*Depression* significantly increases the risk of unmet psychological needs, consistent with previous studies [17, 21, 33]. Symptoms like reduced interest in daily activities may lead to neglect of psychological needs [64]. Early identification and treatment of depression are crucial for addressing this. An evaluation by Miranda‐Castillo et al. confirmed that many unmet needs in the psychological area occur in participants *living alone* and highlighted the need for interventions that involve people with dementia in the community to improve their quality of life in the long term [20]. Higher *education* [17, 56] and access to a *care grade* reduce unmet psychological needs, as they provide better access to support services. Additionally, poor *physical health* and a lack of physical inactivity negatively impact mental well‐being [65, 66], with physical deterioration increasing psychological needs, which often go unmet. The link between older *age* and fewer unmet mental health needs may stem from greater acceptance and adaptation to the disease over time, leading to better coping and reduced psychological needs. Interestingly, an increase in BMI is linked to fewer psychological unmet needs, as studies suggest obesity in late life may be a protective factor against dementia, possibly due to the resources it provides to the brain in old age (from the age of 74) [67].

#### 4.1.4 Social Unmet Needs

Regular *visits to a GP* are associated with fewer unmet social needs [24]. A positive doctor‐patient relationship can influence patient satisfaction [68], and GPs often provide holistic care and social support, helping connect patients to services that meet social and informational needs. In contrast, recent *visits to neurologists* tend to be associated with more unmet social needs, possibly due to the specialized and more stressful nature of these visits. Delivering neurological diagnoses can raise awareness of the illness, potentially leading to stigmatization, feelings of shame, and subsequent social withdrawal among people with dementia. Better *cognition* paradoxically linked to more unmet social needs, perhaps because those with mild impairments overlook their social needs. Lack of a *care grade* and feelings of *loneliness* further impact social functioning. To combat social isolation, early interventions such as community activities, senior groups, or digital technologies can help maintain social connections. These approaches are especially important for individuals with limited mobility [69]. *Female* participants, in particular, experience higher levels of unmet social needs. They are more likely to live alone in old age [70], which limits social interaction and requires more effort to meet social needs. Integrating (single) women into the community could help address this.

## 5 Conclusion

Addressing unmet needs in people with dementia requires a holistic approach that goes beyond physical health. A comprehensive understanding of their environment, mental health, and social connections is essential for effectively addressing unmet needs [14]. The study highlights gaps in community care services, including daytime activities, self‐care, and household support, emphasizing the need for early identification and referral to daycare, home care, or specialized services. Specially qualified professionals like dementia care managers [71] could be vital in both identifying and addressing unmet needs beyond medical and nursing care [11].

Some unmet care needs can be addressed through professional interventions such as applying for long‐term care, regular GP visits, monitoring health indicators like BMI, depression screening, and medication management. However, psychosocial factors such as living alone, loneliness and lack of social support are harder to resolve through medical care alone and are crucial contributors to unmet needs. Promoting social engagement to combat loneliness can significantly improve quality of life and reduce unmet care needs [20]. This highlights the importance of psychosocial interventions in addressing these challenges [27]. Particularly in rural regions, where a large proportion of the sample lives, with long distances and limited healthcare access, these circumstances can hinder adequate medical care and social interaction in old age. Addressing these dual challenges is essential for improving long‐term care outcomes for people with dementia. A longitudinal study would clarify how stress factors (caregiver burden, social isolation, or physical health deterioration) relate to unmet needs over time, capturing dynamic changes as dementia progresses. This could be structured as a cohort study or as a randomized controlled trial, which could test specific interventions aimed at reducing unmet needs. Additionally, investigating the role of technology and social support systems in improving care outcomes, particularly in rural areas, could provide valuable insights for enhancing quality of life for people with dementia and their caregivers.

## 6 Limitations

People with dementia in this study were recruited from networks of physicians who may have better communication standards and more effective cooperation structures, and people with dementia may benefit from these structures (dementia‐specific medications, rapid referrals) [72]. Hence, there may be lower unmet needs in this study sample compared to studies with people with dementia recruited from separate practices or specialists. For this reason, the generalizability of the results may be limited. Another limitation of the study is that this report is based on cross‐sectional data that does not allow for establishing causal relationships between variables. Much of the data was based on self‐reported information from caregivers and PlwD. This could lead to bias as some needs may have yet to be fully captured or misinterpreted. The study also tended to include people with mild to moderate dementia, and it is, therefore, possible that the number of unmet needs among PlwD was underestimated, as people with severe dementia (15%) tended to participate less frequently in the study due to their advanced stage of the disease. Another limitation of our study is that certain characteristics of people living with dementia (PlwD), such as ethnicity, role of carer characteristics or the geographical and emotional closeness of family caregivers, were considered due to lack of availability. Therefore, further qualitative and quantitative research is needed to explore the effects of these factors on unmet and met healthcare needs.

## Conflicts of Interest

The authors declare no conflicts of interest.

## Supporting information

Supporting Information S1

## Data Availability

The data that support the findings of this study are available from the corresponding author upon reasonable request.

## Endnotes

^1^
The long‐term care insurance provider in Germany uses five care grades to rate how independently a person can manage their everyday life and how severely affected their mental, psychological and physical abilities are. The greater the impairment, the higher the care grade and also the long‐term care insurance benefits.
