# Supplementary material for: Identifying and Addressing Unmet Needs in Dementia: The Role of Care Access and Psychosocial Support
Source: Int J Geriatr Psychiatry. 2025 Mar 27;40(4):e70066. doi: 10.1002/gps.70066 (PMC11949772; doi:10.1002/gps.70066)
Supplement: Supplementary file 1 — Supporting Information S1 [file GPS-40-e70066-s001.docx]

**Supplementary Table 1:** Logistic regression for differences between people with dementia without needs vs. those with needs

|  | **Excluded** | | | | | | | |
| --- | --- | --- | --- | --- | --- | --- | --- | --- |
|  | **Environmental needs^1^**  Lost because of no needs = 50 | | **Physical needs^2^**  Lost because of no needs = 19 | | **Psycological needs^2^**  Lost because of no needs = 63 | | **Social needs**  Lost because of no needs = 116 | |
|  | OR (95%-CI) | p-value | OR (95%-CI) | p-value | OR (95%-CI) | p-value | OR (95%-CI) | p-value |
| Age | 1.02 (0.97 – 1.07) | 0.302 | 1.05 (0.98 – 1.12) | 0.151 | **0.94 (0.89 – 0.98)** | **0.011** | 0.98 (0.94 – 1.01) | 0.285 |
| Sex (Ref. female) | 0.66 (0.33 – 1.31) | 0.239 | 0.64 (0.21 – 1.93) | 0.431 | 0.77 (0.41 – 1.44) | 0.417 | 0.87 (0.52 – 1.45) | 0.614 |
| Informal caregiver (Ref. available) | 0.75 (0.25 – 2.28) | 0.624 | 0.45 (0.09 – 2.07) | 0.309 | 0.74 (0.25 – 2.20) | 0.597 | 0.68 (0.28 – 1.63) | 0.394 |
| Living situation (Ref. not alone) | 1.25 (0.65 – 2.41) | 0.498 | 1.93 (0.68 – 5.48) | 0.214 | 0.88 (0.48 – 1.60) | 0.677 | 1.20 (0.73 – 1.99) | 0.461 |
| Cognitive impairment (MMSE) | 0.99 (0.95 – 1.05) | 0.993 | 1.03 (0.95 – 1.11) | 0.435 | 0.99 (0.95 – 1.04) | 0.966 | **0.94 (0.90 – 0.98)** | **0.005** |
| Depression (GDS) | 1.11 (0.95 – 1.30) | 0.157 | 1.32 (0.97 – 1.79) | 0.074 | **1.19 (1.02 – 1.39)** | **0.020** | 1.03 (0.93 – 1.14) | 0.471 |
| Activities in daily living (B-ADL)  Quality of life (EQ-5D-5L) | **1.26 (1.07 – 1.48)**  0.14 (0.01 – 1.06) | **0.004**  0.063 | **1.40 (1.10 – 1.85)**  0.09 (0.01 – 3.54) | **0.007**  0.201 | 1.02 (0.89 – 1.18)  0.22 (0.04 – 1.19) | 0.684  0.080 | **1.26 (1.21 – 1.42)**  0.38 (0.11 – 1.33) | **0.000**  0.132 |

Model^1^: n= 417, Pseudo R^2^: 0.0986, p=0,0002; Model^2^: n= 417, Pseudo R^2^: 0.1790, p=0,0005; Model^3^: n= 417, Pseudo R^2^: 0.0771, p=0,0006; Model^4^: n= 417, Pseudo R^2^: 0.1127, p<0,0001.

**Abbreviations:** OR, odds ratios; Ref, reference; CI, confidence interval; MMSE: Mini Mental State Examination, range 0-30, higher score indicates better cognitive functioning; B-ADL: Bayer Activities of Daily Living Scale, range 0-10, lower score indicates better performance; GDS: Geriatric Depression Scale, sum score 0-15, score≥5 indicates depression; F-SozU: range 0-5; higher score indicates better social support; EQ-5D-5L: European Quality of Life 5 Dimensions 5 Level Version, range 0-1; higher score indicates better health-related quality of life**.**

**Supplementary Table 2:** Poisson Regression models for the number of unmet needs (sensitivity analyses)

|  | **Unmet needs (CANE)** | | | | | | | |
| --- | --- | --- | --- | --- | --- | --- | --- | --- |
|  | **Environmental^1^** | | **Physical^2^** | | **Psychological^3^** | | **Social^4^** | |
|  | **OR** | **CI** | **OR** | **CI** | **OR** | **CI** | **OR** | **CI** |
| Patient age (years) | **.95*** | **.92 - .99** | 1.01 | .97 – 1.04 | **.96*** | **.92 - .99** | .96 | .92 – 1.01 |
| Patient sex (Ref. male) | 1.29 | .76 – 2.20 | 1.10 | .68 – 1.78 | 1.27 | .77 – 2.11 | **1.88*** | **1.05 - 3.37** |
| Living alone (Ref. not alone) | .87 | .51 – 1.51 | 1.54 | .94 – 2.51 | **1.73*** | **1.04 - 2.86** | 1.42 | .79 – 2.53 |
| Caregiver availability (Ref. yes) | 1.38 | .49 – 3.87 | **2.81*** | **1.03 - 7.64** | .75 | .27 – 2.02 | 1.94 | .59 – 6.39 |
| Education (Ref. Higher sec. education) | 1.66 | .98 – 2.83 | **1.72*** | **1.06 - 2.78** | **1.86*** | **1.12 - 3.08** | 1.33 | .75 – 2.37 |
| Financial situation (Ref. good) | 1.40 | .71 – 2.64 | .94 | .52 – 1.71 | 1.65 | .91 – 3.01 | 1.25 | .61 – 2.56 |
| GP visit last 3 months (Ref. no) | 1.44 | .65 – 3.14 | 1.12 | .54 – 2.30 | 1.22 | .57 – 2.60 | .58 | .26 – 1.30 |
| Neurologists visit last 3 months (Ref. no) | .68 | .39 – 1.20 | 1.11 | .67 – 1.83 | 1.06 | .63 – 1.78 | 1.63 | .89 – 2.96 |
| Functional impairment (B-ADL) (Ref. good) |  |  |  |  |  |  |  |  |
| Average | 1.08 | .51 – 2.29 | **2.55*** | **1.24 - 5.25** | 1.67 | .81 – 3.46 | 1.11 | .43 – 2.83 |
| Poor | 1.63 | .64 – 4.18 | **8.40***** | **3.39-20.81** | **2.82*** | **1.15 - 6.93** | 1.98 | .67 – 5.83 |
| Depression (GDS) (Ref. no depressive symptoms) |  |  |  |  |  |  |  |  |
| Indication for depression | 1.68 | .85 – 3.31 | 1.74 | .91 – 3.31 | **2.13*** | **1.11 - 4.08** | 1.73 | .81 – 3.68 |
| Cognitive impairment (MMSE) (Ref. mild) |  |  |  |  |  |  |  |  |
| Moderate/ severe | .85 | .49 – 1.48 | **.58*** | **.35 - .97** | 1.01 | .59 – 1.69 | .82 | .45 – 1.50 |
| General health (EQ-5D-5L index) (Ref. good) |  |  |  |  |  |  |  |  |
| Average | .84 | .44 – 1.60 | 1.24 | .70 – 2.22 | .85 | .46 – 1.54 | 1.77 | .88 – 3.56 |
| Poor | 1.03 | .44 – 2.37 | 1.18 | .54 – 2.58 | .61 | .27 – 1.38 | .97 | .37 – 2.53 |
| Body Mass Index (BMI, Kg/m^2^) (Ref. normal weight) |  |  |  |  |  |  |  |  |
| Underweight/ Pre-obesity+Obesity | .55 | .33 - .91 | .72 | .45 – 1.15 | .76 | .46 – 1.23 | **.54*** | **.31 - .95** |
| Having a care grade (Ref. yes) | **3.38***** | **1.95 - 5.87** | **3.02***** | **1.81 - 5.04** | **2.50***** | **1.48 - 4.24** | **4.66***** | **2.48 - 8.73** |
| Number of diagnoses | **.95*** | **.91 - .98** | .99 | .96 – 1.02 | .97 | .94 – 1.01 | 1.01 | .98 – 1.04 |
| Number of drugs taken | **1.10**** | **1.02 - 1.19** | 1.05 | .98 – 1.13 | 1.03 | .96 – 1.11 | 1.01 | .92 – 1.09 |
| Social support (F-SozU) (Ref. high) |  |  |  |  |  |  |  |  |
| Low/Average | .74 | .42 – 1.30 | **1.71*** | **1.02 - 2.84** | 1.26 | .74 – 2.14 | 1.43 | .78 – 2.61 |
| Loneliness (Ref. not lonely) |  |  |  |  |  |  |  |  |
| Moderate/ Severe | **2.51**** | **1.44 - 4.36** | .89 | .54 – 1.48 | 1.34 | .80 – 2.24 | 1.48 | .84 – 2.62 |

Model^1^: n=367, Pseudo R^2^=0.1245, p<0.0001; Model^2^: n=354, Pseudo R^2^= 0.0994, p=0.0004; Model^3^: n=398, Pseudo R^2^ = 0.1297, p<0.0001; Model^4^: n=301, Pseudo R^2^ = 0.1672, p<0.0001. p*<0.05; p**<0.01; p***<0.001 tested for each independent variable with the dependent variable (CANE).

**Abbreviations:** MMSE: Mini Mental State Examination, range 0-30, higher score indicates better cognitive functioning (categories: 29-20 mild, 19-10 moderate, ≤9 severe); B-ADL: Bayer Activities of Daily Living Scale, range 0-10, lower score indicates better performance (categories: <3 good, 3-<8 average, 8-10 poor); GDS: Geriatric Depression Scale, sum score 0-15, score≥5 indicates depression (categories: 0-5 no depressive symptoms, >6 depression); F-SozU: Perceived social support questionnaire, range 0-5; higher score indicates better social support (categories: <3 low, 3-<4 average, >4 high); EQ-5D-5L; range 0-1; higher score indicates better health-related quality of life (categories: >0.9 good, ≤0.9-0.5 average, ≤0.5 poor); Loneliness: range 0-11; higher score indicates severe loneliness (categories: 0-2 not lonely, 3-8 moderate, >9 severe). SD: Standard Deviation. OR=Odds Ratio; CI=95% Confidence Interval; Ref=reference.
